# A homozygous G insertion in MPLKIP leads to TTDN1 with the hypergonadotropic hypogonadism symptom

**DOI:** 10.1186/s12881-018-0723-5

**Published:** 2018-12-31

**Authors:** Yi-Kun Zhou, Xiao-Chun Yang, Yang Cao, Heng Su, Li Liu, Zhi Liang, Yun Zheng

**Affiliations:** 1 Department of Endocrinology and Metabolism, First People’s Hospital of Yunnan Province (The Affiliated Hospital of Kunming University of Science and Technology), Kunming, 650032 People’s Republic of China; 2 Department of Ophthalmology, First People’s Hospital of Yunnan Province (The Kunhua Affiliated Hospital of Kunming University of Science and Technology), Kunming, 650032 People’s Republic of China; 3 Center of Growth, Metabolism and Aging, Key Lab of Bio-Resources and Eco-Environment of Ministry of Education, College of Life Sciences, Sichuan University, Chengdu, 610064 People’s Republic of China; 4 Yunnan Key Laboratory of Primate Biomedical Research, Institute of Primate Translational Medicine, Kunming University of Science and Technology, Kunming, 650500 People’s Republic of China; 5 Department of Information center, First People’s Hospital of Yunnan Province (The Affiliated Hospital of Kunming University of Science and Technology), Kunming, 650032 People’s Republic of China

**Keywords:** Trichothiodystrophy nonphotosensitive 1 (TTDN1), Hypergonadotropic hypogonadism, MPLKIP, Mutation, Small Indel

## Abstract

**Background:**

Trichothiodystrophy nonphotosensitive 1 (TTDN1) is a disease with mental retardation, brittle hair. Some cases of the diseases are caused by mutations of the MPLKIP gene.

**Methods:**

We carefully identified the clinic characteristics, the sulfur level and pattern of the hair shafts of a female patient of with the symptom of hypergonadotropic hypogonadism, and of her parents and brother whose are healthy. We also collected the blood sample of the patient and performed the exon sequencing. One G insertion in MPLKIP was identified after analyzing the obtained exon sequencing profile. The G insertion sites in the patient, her parents and brother, were verified using Sanger sequencing. The G insertion in MPLKIP were compared to the dbSNP.

**Results:**

The female patient of TTDN1 carries a homozygous G insertion (rs747470385) in the MPLKIP gene. The parents and brother of the patient are heterozygous carriers of the same mutation, but are healthy. The hair shafts of the patient had a tiger-tail pattern with relatively low sulfur levels. To the best of our knowledge, this is the first report that autosomal recessive inheritance of the G insertion in the MPLKIP gene results in TTDN1.

**Conclusion:**

Our results indicate that the homozygotic G insertion in MPLKIP results in the TTDN1 with hypergonadotropic hypogonadism, while heterozygous carriers of the same mutation have no symptoms and healthy. These results provide novel insights into the association of mutations in MPLKIP and TTDN1 with hypergonadotropic hypogonadism.

## Background

Trichothiodystrophy (TTD) is an autosomal recessive disorder of neuroectodermal origin. This condition is characterized by a cross-banding pattern, i.e. tiger-tail, under polarized light and low sulfur content in the hair shafts, which are associated with variable and neuroectodermal symptoms. The condition manifests with brittle hair, intellectual disability, dwarfism, decreased fertility, microcephaly, abnormal facial features, ichthyosis premature aging, nail dystrophies, and a propensity for respiratory infections [1]. TTD is divided into two types: photosensitive (TTD1-3) and nonphotosensitive (TTD4 or TTDN1). Photosensitivity exists in about 50% of TTD patients, which is due to reduced amounts of transcription/repair factor IIH involved in global genome and transcription repair [2, 3]. Mutations in the MPLKIP gene cause some cases of TTDN1.

MPLKIP is located on chromosome 7p14.1 and includes two coding exons that encode a protein called M-phase-specific PLK1-interacting protein, which can interact with PLK1 protein. Based on the function of the PLK1 protein and its location in the nucleus, the MPLKIP protein plays a role in regulating the cell cycle. It is ubiquitously expressed in epidermis, hair follicles, brain, heart, liver, kidney, skeletal muscle, pancreas, lung, and placenta [4–6]. The precise function of MPLKIP has not yet been determined, but clinical symptoms of TTDN1 result from mutation of the gene [4].

## Results

### Physical characteristics of the patient

A 16 year old girl in China with primary amenorrhea and mental retardation was sent to the endocrinology clinic by her parents. The patient was born full term via spontaneous vaginal delivery. Her birth weight and length are unknown. Her parents are cousins related by blood. When the patient was 7 years old in primary school, she was observed to have significant mental retardation. Patients had no complaints of tetany, ostalgia, hearing loss, or hyposmia. Menarche did not occur until 16 years of age. A small amount of bleeding due to an artificial menstrual cycle induced by local doctors occurred for 5 months. The heights of her father and mother were 171 and 154 cm, respectively. The development and intelligence of her elder brother was normal. There was no history of any endocrine illness in other family members.

Upon physical examination, her blood pressure was 100/66 mmHg, temperature 36 ^∘^C, pulse rate 82 beats/min, and respiratory rate 20 breaths/min (see Table 1). The patient had a height of 150 cm (<3rd centile on the 2009 Chinese Academy of Pediatrics growth chart) with a weight of 39 kg (Table 1). Arm span was 145 cm with an upper/lower segment ratio of 0.9:1 (Table 1). There was a lack of secondary sexual characteristics Tanner BII and PH I (Table 1). Bilateral cataracts were found (Table 1). The oral cavity had no enamel hypoplasia. The neck was not short with excess skin and no goiter. There were no notable special facial features except sparse eyebrows and brittle hair. Abnormal forearm carrying angles were not found. The fifth middle phalanxes on their both hands were short. Chvostek’s sign, Trousseau’s sign, and signs of neuromuscular irritability were not present. Her skin was normal with no UV light sensitivity or ichthyosis issues.

**Table 1:** The physical characteristics of the patient

Characteristics	Value	Characteristics	Value
Age (year)	16	BMD^b^	Z: -4.5
Gender	Female	FSH^b^ (3.5-12.5mIU/ml)	127.4
Height (cm)	150^a^	LH^b^ (2.4-12.6 mIU/ml)	65.25
Weight (kg)	39	E2^b^ (45.4-854 pmol/l)	18.35
Blood Pressure (mmHg)	100/66	25 (OH) D3 (ng/ml)	19.68
Hair shaft	Tiger-tail pattern	Intelligence	<40^c^
Pubes	Tanner I	Skeletal age (year)	12
Breast	Tanner II	The same symptoms in family	No
Cataracts	Bilateral	Family history	Her parents are cousins
Upper/lower	0.9:1	Sulfur level of hair	Lower than normal^d^
Menstruation	primary amenorrhea	Two stimulation tests for GH^b^	Normal

^a^The value is smaller than the 3rd centile on the 2009 Chinese Academy of Pediatrics Growth Chart

^b^Abbreviations are BMD: Bone Mineral Density, FSH: Follicle-Stimulating Hormone, LH: Luteal hormone, E2: Estradiol, and GH: Growth Hormone

^c^Value was based on Wechsler intelligence scale

^d^The value of patient is 1.905 mg/kg, which is significantly lower than that of normal group, i.e., mother: 4.177 mg/kg, father: 4.811 mg/kg, and a normal control: 4.62 mg/kg (*P*=0.02, *t*-test)

Testing revealed a follicle-stimulating hormone (FSH) of 127.4 mIU/ml (normal range: 3.5-12.5 mIU/ml), progesterone (PROG): 0.52 nmol/l (0.6-4.7 nmol/l), luteal hormone (LH): 65.25 mIU/ml (2.4-12.6 mIU/ml), estradiol (E2) 18.35 pmol/l (45.4-854 pmol/l), prolactin (PRL): 17.3 ng/ml (3.4-24.1 ng/ml) and testosterone (TEST): 0.18 nmol/ml (0.16-1.33 nmol/ml) (see Table 1). Serum 25 hydroxy vitamin D was 19.68 ng/ml (>30 ng/ml). Both arginine and levodopa stimulation tests for growth hormone were carried out, revealing normal growth hormone responses to stimulation. Liver, renal, thyroid, coagulation, fasting plasma glucose, blood fat, and dynamic cortisol level tests were normal. The serum alkaline phosphatase, calcium, phosphorus, 24 hr urinary calcium, and parathormone were within reference ranges. However, the 24 hr urinary phosphorus was 5.99 mmol/d (12.9-42 mmol/d).

**Fig. 1:**
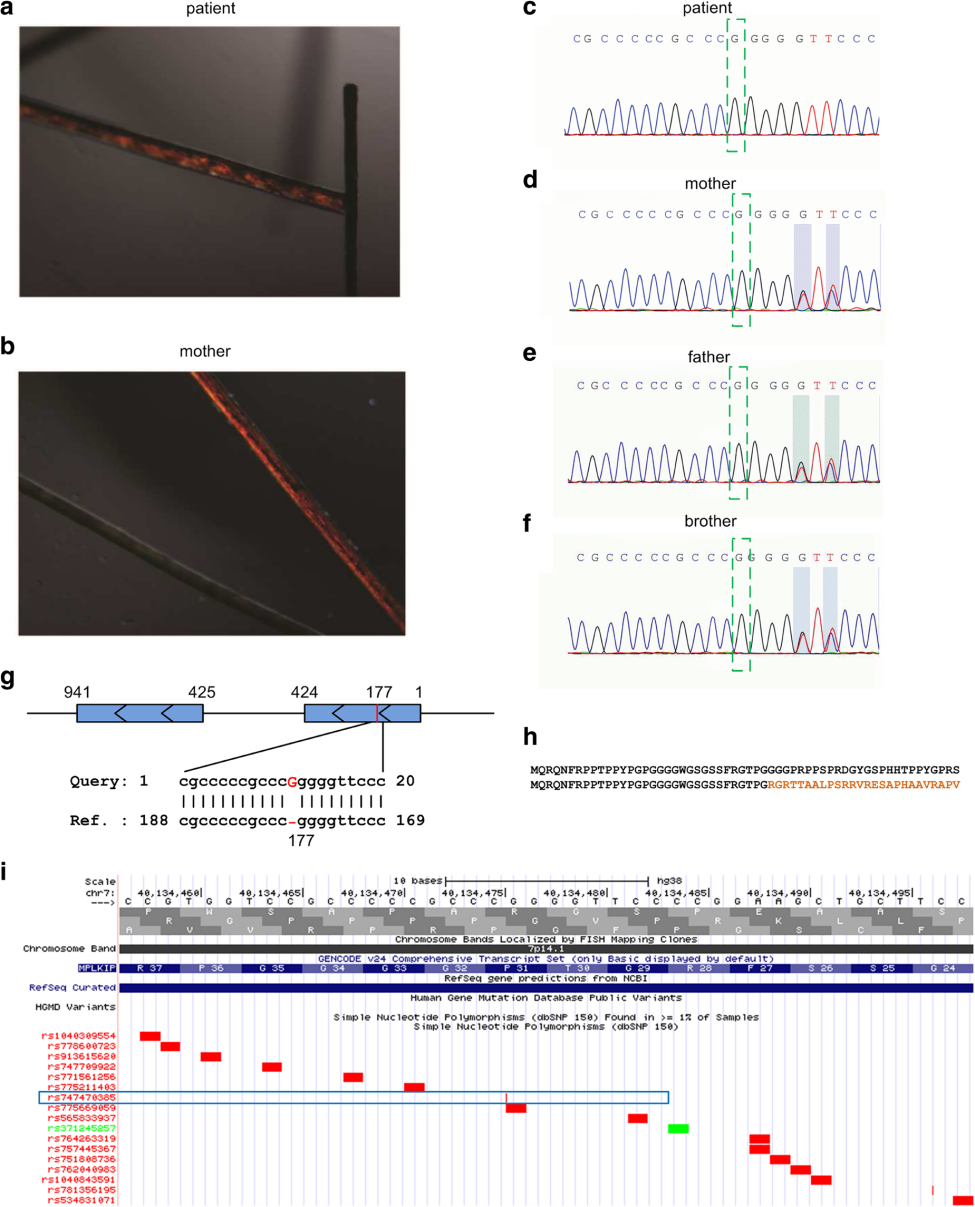
The brittle hair of the patient and G insertion identified in the patient. **a** The typical alternating dark and light “tiger tail” banding was seen by polarized light microscopy in hair of the patient. **b** The typical alternating dark and light “tiger tail” banding was not seen by polarized light microscopy in hair of the patient’s mother. **c** to **f** The Sanger sequencing of the mutated region in MPLKIP for the patient, her mother, her father and her elder brother, respectively. **g** The position of the G insertion in the MPLKIP gene. Query is the sequence obtained in Sanger sequencing. Ref. is the sequence of MPLKIP mRNA (NM_138701.3). **h** The amino acid sequences of wild type MPLKIP gene (top) and the mutated MPLKIP gene with the identified G insertion. Only the first 58 amino acids of the wild type MPLKIP protein are shown. **i** The G insertion was examined in UCSC Genome Browser to be compared to the dbSNP (v150). The G insertion in MPLKIP is rs747470385 (-/G), as indicated by the blue rectangle

MRI of the hypophysis was normal. X-rays of the wrist and hand suggested that skeletal age was around 12 years, which did not correspond to chronological age. Double energy x-ray bone density suggested osteoporosis (Z: -4.5). Wechsler intelligence scale revealed her intelligence quotient was lower than 40 points. Electrocardiogram showed sinus rhythm, and first degree a-v block and ultrasonic cardiogram were normal. B ultrasonic examination showed that Liver, bile, pancreas, spleen and kidney were normal and both the uterus (43 mm ×28 mm ×15 mm) and ovaries (right 16 mm ×5 mm, left 14 mm ×4.5 mm) were smaller and the endometrium was thin. Her hair shafts were examined by polarized light microscopy and had a characteristic tiger-tail pattern (Fig. 1a), which was not noticed in her mother’s hair (Fig. 1b). The sulfur level of the hair measured by atomic emission spectrometry was 1.905 mg/kg significantly lower than both parents and a healthy control (patient: 1.905 mg/kg compared to mother: 4.177 mg/kg, father: 4.811 mg/kg, and a normal control: 4.62 mg/kg) (*P* = 0.02, *t*-test).

### Characterizing the genomic mutations of the patient

Chromosomal karyotyping revealed the patient had a normal female karyotype (46, XX). In order to acquire a molecular diagnosis, exon sequencing was performed for the patient. After analyzing the exon sequencing profile, we found a G insertion in the first exon of the MPLKIP gene. We then confirmed the G insertion with Sanger sequencing, for the patient, and her father, mother, and elder brother (see green boxes in Fig. 1c to f). From Fig. 1c, it could be seen that the Sanger sequencing of the patient is clear and has no minor peaks in the region. However, in the Sanger sequencing results of her mother, father and brother, two nucleotides after the G insertion sites (in the shaded regions of Fig. 1d to f, respectively) have minor or almost equal peaks, suggesting heterozygous genotypes. To summarize, the patient is homozygous for the G insertion, while her father, mother, and brother are heterozygous carriers (see Fig. 1d to f).

We carefully examined the G insertion in MPLKIP and found that the G insertion locates immediately after the 177th nucleotide in MPLKIP transcript (Fig. 1g). The normal MPLKIP protein consists of 179 amino acids. The G insertion leads to a significantly short amino acid sequence with only 58 residuals, and the sequence changes after the 32nd amino acid (see Fig. 1h). Thus, the homozygous G insertion may lead to total deletion of functional MPLKIP protein in the patient. We compared the identified G insertion to the latest dbSNP (v150) using UCSC Genome Browser and found that the G insertion is a reported SNP, rs747470385 (-/G) (Fig. 1i).

### The protein-protein interaction network of MPLKIP

**Fig. 2:**
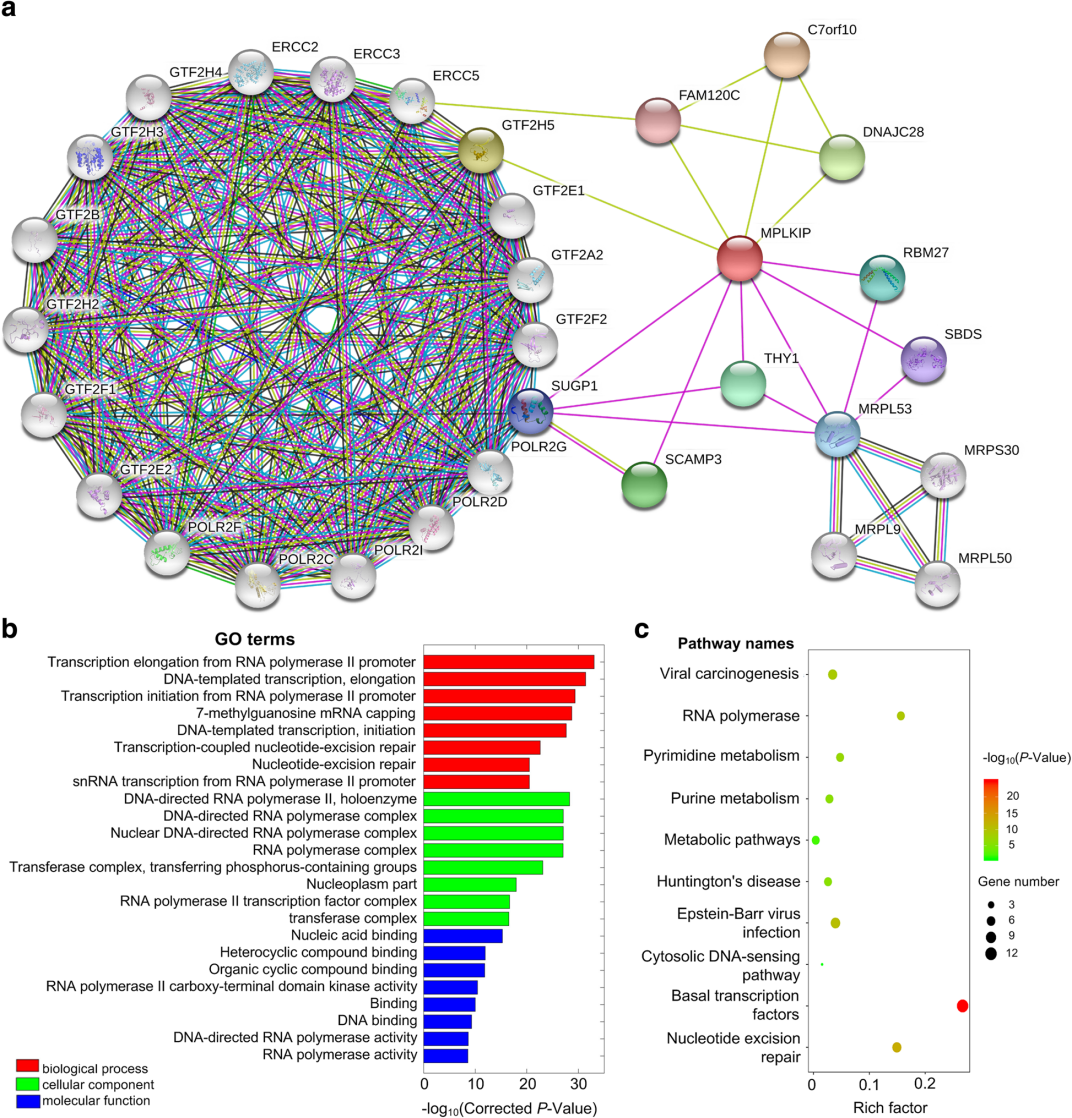
The protein-protein interaction network of MPLKIP. **a** The proteins interacting with MPLKIP examined using STRING [22]. An edge between two proteins (shown as nodes) means these two proteins are interacting proteins. Purple and azure edges stand for experimentally determined interactions and interactions from curated databases, respectively. Green, red, and blue edges stand for predicted interactions from gene neighborhood, gene fusions and gene co-occurrence, respectively. Light green and black edges stand for other interactions from text mining and co-expression, respectively. **b** Some of the enriched GO terms for genes in Part (**a**). **c** The enriched KEGG pathways for genes in Part (**a**)

We investigated the functional roles of MPLKIP by examining its protein-protein interaction network as well. As shown in Fig. 2a, MPLKIP serves as a bridge between the RNA Polymerase II complex (left part) and many other co-factors. Among proteins interacting with MPLKIP, SBDS encodes a highly conserved protein that plays an essential role in ribosome biogenesis. SBDS protein interacts with elongation factor-like GTPase 1 to disassociate eukaryotic initiation factor 6 from the late cytoplasmic pre-60S ribosomal subunit allowing assembly of the 80S subunit. Mutations within SBDS are associated with the autosomal recessive disorder Shwachman-Bodian-Diamond syndrome (or Shwachman-Diamond syndrome), which is a rare congenital disorder characterized by exocrine pancreatic insufficiency, bone marrow dysfunction, skeletal abnormalities and short stature. FAM120C encodes a potential transmembrane protein and lies in a region where mutations and deletions have been associated with intellectual disability and autism. Some of the symptoms caused by mutations in SBDS and FAM120C, such as short stature and intellectual disability, are similar to shown in our patient.

We then performed Gene Onotology (GO) and KEGG pathway enrichment analysis for the interacting proteins of MPLKIP in Fig. 2a using KOBAS2 [7]. As shown in Fig. 2b, these genes are significantly enriched in many RNA Polymerase II related transcription GO terms, such as Transcription elongation from RNA polymerase II promoter, DNA-templated transcription, elongation, and Transcription initiation from RNA polymerase II promoter, DNA-directed RNA polymerase II, holoenzyme, DNA-directed RNA polymerase complex, Nucleic acid binding, and RNA polymerase II carboxy-terminal domain kinase activity. In Fig. 2c, it is shown that these genes are enriched in transcription related pathways too, such as Basal transcription factors and Nucleotide excision repair. Mutations in genes on the nucleotide excision repair pathway are associated with diseases, such as xeroderma pigmentosum (XP), Cockayne syndrome (CS) and trichothiodystrophy (TTD). In summary, these results suggest that the loss of MPLKIP caused by the G insertion will disrupt the interactions between many other co-factors and Pol II complex, and affect the Pol II involved transcription activities, which contributed to symptoms witnessed in the patient.

### Treatments and consequences of the patients

Due to gonadal failure, the patient was initially given 0.5 mg estradiol valerate once a day. Low-dose oestrogen monotherapy enabled normal breast and uterine development. She had a vitamin D deficiency (serum 25 hydroxy vitamin D was 19.68 ng/ml) with osteoporosis, although osteoporosis in this patient was probably one of the phenotypes of hypogonadism; therefore, oral calcitriol and calcium carbonate were given at doses of 0.25 *μ*g and 600 mg, respectively, once daily. Although she had a short stature, her parents did not want to treat the problem, so no treatments were given to correct her height.

The patient followed up with us after 3 months. Her breast developed to Tanner BIII and B ultrasonic examination showed both her uterus (61 mm ×42 mm ×26 mm) and ovaries (right one was 25 mm ×16 mm, while the left one was not clear) were bigger than before and the thickness of endometrium went from thin to 3 mm with estradiol increased to 330.6 pmol/L (normal range 45.4-854 pmol/L). Her bone mineral density did not increase with initial estradiol valerate treatment and a short treatment course with calcitriol. The patient was kept on oestrogen and vitamin D supplements and was advised to follow-up again after 3 months.

## Discussion

The patient had a number of symptoms associated with endocrine disorders, including hypergonadotropic hypogonadism, short stature, and osteoporosis. Meanwhile, she presented with distinct characteristics of mental retardation, brittle hair with a tiger-tail pattern and low sulfur level, and cataract without photosensitivity. Many cases reported that TTDN1 manifested decreased fertility but hypergonadotropic hypogonadism confirmed ovarian lesions were the reason of gonad disorder in this paper.

The relationship between TTDN1 and MPLKIP was reported in 2005 [4]. Only 14 cases were reported in [8–12] after the study of Nakabayashi et al. [4]. As summarized in Table 2, there are different special phenotypic manifestations in addition to common symptoms that derive from various genetic mutations of MPLKIP. The homozygous splice mutation c.339 + 1G > A within MPLKIP results in mitral regurgitation [8]. Heller et al. [10] reported that mutations at two different loci cause autism spectrum disorder (see Table 2). Mutation c. 277delT results in seizure disorders [10], and mutation c.505dupA within the MPLKIP gene has been shown to cause severe renal failure [9]. Swagemakers et al. [13] reported that Pollitt syndrome patients, with mental and physical retardation and trichorrhexis nodosa, carry C326delA. Botta et al. [11] reported several cases of axial hypotonia and reduced motor coordination due to deletions at four different loci, and several cases with failure to thrive, very poor motor performances and speech due to deletions at two different loci (see Table 1). Nakabayashi et al. [4] and Przedborski et al. [14] reported that 187_188delGG causes ataxia.

**Table 2:** A summary of reported patients with mutations in the MPLKIP gene and clinical features

Country	Variation	Type	PS	HA	FD	MR	HG	O/O	CT	SS	Particularity	Ref.
Pakistan	c.339 +1G > A	Splice	N	Y	Y	Y	NA	NA	Y	Y	Mitral regurgitation	[7]
Caucasian	c.2T>C (initiation codon)		N	Y	Y	Y	NA	Y	N	N	Autism spectrum disorder	[9]
Caucasian	Deletion of ∼120kb; c.227delG	Deletion	N	Y	Y	Y	NA	Y	Y	Y	Aortic arch with aberrant left subclavian artery	[9]
Caucasian	c.277delT; Deletion of ∼92 kb	Deletion	N	Y	Y	NA	NA	NA	NA	Y	Atrial septal defect; pulmonic stenosis	[9]
Caucasian	4 bp insertion & deletion of ∼5kb starting at c.279	Insertion & deletion	Y	Y	Y	Y	NA	NA	NA	Y	Autism spectrum disorder; epilepsy with grand mal seizures	[9]
Israel	c.505dupA mutation	Duplication	N	Y	NA	Y	Y	NA	N		Renal failure, splenomegaly	[8]
Netherlands	c.326delA	Deletion	NA?	Y?	NA	Y?	NA	NA	NA	NA	NA	[12]
Italy	Deletion of 11-31kb	Deletion	N	Y	NA	NA	NA	NA	NA	Y	Axial hypotonia and reduced motor coordination	[10]
Italy	c.148_152delCAC AC	Deletion	N	Y	NA	Y	NA	NA	NA	Y	Failure to thrive, very poor motor performances and speech	[10]
Italy	c.277delTc.148_15 2delCACAC	Deletion	N	Y	NA	NA	NA	NA	NA	Y	Axial hypotonia and reduced motor coordination	[10]
Kuwait (Indian)	c.229delC	Deletion	N	Y	NA	Y	NA	NA	NA	Y	Failure to thrive, very poor motor performances and speech	[10]
Iraq	Deletion of at least 150 kb	Deletion	N	Y	NA	NA	NA	NA	NA	Y	Axial hypotonia and reduced motor coordination	[10]
Netherlands	c.277delT	Deletion	N	Y	NA	NA	NA	NA	NA	Y	Axial hypotonia and reduced motor coordination	[10]
Italy	partial exon 1 and entire exon 2	Deletion	N	Y	NA	Y	NA	NA	NA	Y	Severe nervous system impairment	[5]
Morocco	187_188delGG	Deletion	N	Y	Y	Y	Y	Y	NA	Y	Ataxia	[5, 13]
Amish	c.480A>G	A ->G	NA	Y	NA	Y	Y	NA	NA	Y		[5]
Peru	{arr[hg19]7p14.1 (40,140,770-40,265,451)x0}	a 125 kb homozygous deletion	NA	Y	Y	Y	NA	NA	NA	Y	Glutaric Aciduria type 3	[11]
China	rs747470385	-/G	N	Y	N	Y	Y	Y	Y	Y		This study

The abbreviations of the titles of the columns are PS: Photosensitivity, HA: Hair Abnormality, FD: Facial deformity, MR: Mental Retardation, HG: Hypogenadism, O/O: Osteoporosis/Osteopenia, CT: Cataract, and SS: Short Stature

Although the disease with low incidences displays many of the characteristics of endocrine disease, we hope it can be easily recognized by endocrinologists. This is the first report of autosomal recessive inheritance of the G insertion (rs747470385) and provides novel insights into endocrine symptoms associated with hypergonadotropic hypogonadism. Although the relationship between hypogonadism and the MPLKIP gene mutation is unclear, the gene plays a key role in regulating in cytokinesis and mitosis that may be a possible reason to explain the relationship.

TTDN1 should be considered in patients with hypogonadism. Hair shafts should be examined by polarized light microscopy and sulfur levels in the hair can be measured as a simple and convenient method for screening for TTDN1.

The wild-type MPLKIP gene is translated into a protein with 179 residues. Insertion of G at base 177 strongly shifts translation. Some termination codons occur in the mutant, implying the mutant protein may significantly differ from the wild-type protein. There are only short polypeptides of ≤58 residues if translated (see Fig. 1h). The homologous sequence of the mutant was not found in the Uniprot database [15]. For the short polypeptides, their secondary structures [16] and disorder sequence [17] predictions show the majority of the polypeptides are coils. It implies the polypeptides could be very flexible without any stable 3D structures. Therefore, the mutant proteins tend to degrade quickly. Therefore, all clinical symptoms in the patient may result from loss of function of the original protein. To summarize, the homozygous G insertion (rs747470385) in her MPLKIP gene caused trichothiodystrophy nonphotosensitive 1 (TTDN1) with hypergonadotropic hypogonadism.

Our results suggest the homozygous G insertion (rs747470385) could potentially be used as a genetic method to screen patients with hypergonadotropic hypogonadism for their diagnosis as TTDN1.

## Conclusions

The contributions of this work are three folds. First, our results indicate that the homozygotic G insertion (rs747470385) cause TTDN1. Second, the homozygotic G insertion could potentially be used as a diagnostic marker of TTDN1. Third, our results indicate that the heterozygous rs747470385 does not lead to TTDN1. However, the marriage between heterozygous rs747470385 carriers should be prevented to minimize the risk of sick offspring.

## Methods

Physical and clinical examinations were performed for the patient, her parents and her elder brother in the First People’s Hospital of Yunnan Province (The Kunhua Affiliated Hospital of Kunming University of Science and Technology, abbreviated as FPHY). The sulfur level of the hair was examined using inductively coupled plasma-atomic emission spectrometry at Kunming CTER Trace Element Measurement Research Center, Kunming, Yunnan, China.

The blood sample of the patient was collected in FPHY. The DNA sample was extracted and used for DNA subtotal exon sequencing at the RunningGene Inc, Beijing, China. The obtained exon sequencing profile had been deposited into the NCBI SRA database and available with the accession number SRR7427760. The obtained sequencing profile was aligned to human genome (hg38) with Bowtie2 [18]. The mutations were analyzed using SAMTools [19] and BCFTools [20] from the alignment results of Bowtie2.

The G insertion in the patient, her mother, father, and elder brother, were verified using Sanger sequencing with the forward primer of CTGGAGTAGGAGCCAGGGT and reverse primer of GGAGGGCCGGTTGATACAG. After examining results of Sanger sequencing, a homozygous G insertion was found in the first exon of the MPLKIP gene of the patient. Heterozygous G insertions at the same positions were found in the parents and brother of the patient. The obtained G insertion was compared to the latest dbSNP using the UCSC Genome Browser [21].

The protein-protein interaction networks of MPLKIP was examined by using STRING [22]. A minimum required interaction score of 0.4 and the second shell with no more than 20 interactors were used when searching the protein “MPLKIP” in the species of “Homo sapiens” in STRING. The enriched Gene Ontology terms and KEGG pathways of proteins interacting with MPLKIP were analyzed by using KOBAS2 [7].

## Abbreviations

BMD: Bone Mineral Density
CS: Cockayne syndrome
E2: Estradiol
FSH: Follicle-Stimulating Hormone
GH: Growth Hormone
GO: Gene Onotology
LH: Luteal Hormone
MRI: Magnetic Resonance Imaging
PRL: Prolactin
PROG: progesterone
TEST: Testosterone
TTD: trichothiodystrophy
TTDN1: trichothiodystrophy nonphotosensitive 1
XP: xeroderma pigmentosum
